# Fibromyalgia Syndrome: A Case Report on Controlled Remission of Symptoms by a Dietary Strategy

**DOI:** 10.3389/fmed.2018.00094

**Published:** 2018-04-30

**Authors:** Silvia Maria Lattanzio, Francesca Imbesi

**Affiliations:** ^1^Department of Biomedical Sciences, University of Padova, Padova, Italy; ^2^Neurological Department, Ospedale Niguarda Ca’ Granda, Milan, Italy

**Keywords:** fibromyalgia syndrome, fibromyalgia symptom remission, dietary strategy, exclusion diet, chronic pain, fructose withdrawal, tryptophan, serotonin

## Abstract

A 34-year-old woman suffered from significant chronic pain, depression, non-restorative sleep, chronic fatigue, severe morning stiffness, leg cramps, irritable bowel syndrome, hypersensitivity to cold, concentration difficulties, and forgetfulness. Blood tests were negative for rheumatic disorders. The patient was diagnosed with Fibromyalgia syndrome (FMS). Due to the lack of effectiveness of pharmacological therapies in FMS, she approached a novel metabolic proposal for the symptomatic remission. Its core idea is supporting serotonin synthesis by allowing a proper absorption of tryptophan assumed with food, while avoiding, or at least minimizing the presence of interfering non-absorbed molecules, such as fructose and sorbitol. Such a strategy resulted in a rapid improvement of symptoms after only few days on diet, up to the remission of most symptoms in 2 months. Depression, widespread chronic pain, chronic fatigue, non-restorative sleep, morning stiffness, and the majority of the comorbidities remitted. Energy and vitality were recovered by the patient as prior to the onset of the disease, reverting the occupational and social disabilities. The patient episodically challenged herself breaking the dietary protocol leading to its negative test and to the evaluation of its benefit. These breaks correlated with the recurrence of the symptoms, supporting the correctness of the biochemical hypothesis underlying the diet design toward remission of symptoms, but not as a final cure. We propose this as a low risk and accessible therapeutic protocol for the symptomatic remission in FMS with virtually no costs other than those related to vitamin and mineral salt supplements in case of deficiencies. A pilot study is required to further ground this metabolic approach, and to finally evaluate its inclusion in the guidelines for clinical management of FMS.

## 1 Introduction

Fibromyalgia syndrome (FMS) is a challenging, complex, heterogeneous, chronic, and often disabling disorder (1, 2). Its pathophysiology is still poorly understood (1, 3, 4). Chronic musculoskeletal widespread pain, fatigue, non-restorative sleep, mood disturbances, and cognitive impairments characterize this condition (1, 2). Furthermore, a constellation of comorbidities, only apparently not connected, afflicts the patients (2–8), and have a clear common denominator in serotonin (5-HT). The symptoms can range from mild to severe, determining in the worst cases an invalidating condition that dominates daily life (9). They may lead to an occupational and social disability with associated direct and indirect economic costs (10).

Even if pain is the core symptom in FMS, non-painful symptoms may also impact the quality of life of patients. Recognizing those symptoms may be difficult in the absence of other apparent organic diseases. This makes FMS a real challenge for physicians and healthcare professionals.

We report the first case of controlled remission of symptoms in FMS, following a novel metabolic approach. The therapeutic protocol is a strict diet, focused on the withdrawal of food components that may interfere with the absorption of l-tryptophan (Trp), 5-HT precursor (11).

## 2 Case Presentation

### 2.1 Patient Presentation

The patient is a 34-year-old woman, body mass index 18, Caucasian, high level instruction.

### 2.2 Old Past History

The patient’s past history, *ex post* relevant for FMS differential diagnosis, includes irritable bowel syndrome-constipation (IBS-c), bloating, dismenorrhea experienced since adolescence, Raynaud’s phenomenon, trapezium contractures, leg and foot cramps, especially at night or waking up in the morning.

### 2.3 Past History

The onset of lower back pain, restless legs, and morning stiffness occurred few months after a surgery. The symptoms were first described as mild in severity, particularly concerning pain, and erratic. One-year later, lower back pain and hip pain forced the patient to bed rest. Non-steroidal anti-inflammatory drugs (i.e., ibuprofen) and muscle relaxants (i.e., thiocolchicoside) were prescribed by the primary care clinician, and led to mild effects. Magnetic resonance imaging and X-ray investigations revealed a lumbar disk hernia and no lesions or abnormalities at hips. One-year later, during autumn temperatures decrease a further episode of lower back pain and stiffness occurred forcing the patient to bed rest for more than 2 weeks. Previous treatment had no effectiveness and corticosteroid drug (i.e., prednisone) led to no appreciable relief. Pain was in part relieved by gabapentin, but only with slow dynamics and with collateral effects of suicidal thoughts and mental confusion.

Further magnetic resonance and X-ray investigations confirmed the previous diagnosis with no new data to explain relapse worsening. Similar symptoms of lower back pain during the following 2 years were attributed to the same cause. These episodes recurred three to four times a year, affecting life quality and mobility, and forcing patient to bed. Intriguingly, the worst episodes appeared to correlate to decreasing temperatures of the autumn.

### 2.4 Recent Past History

The symptoms increased slightly, but progressively with unpredictable and fluctuating nature. Morning stiffness required more than 40 min to get up while awakening. Fatigue unrelieved by rest, low back pain, migrant aches in the joints, musculoskeletal widespread pain, short-term memory loss, concentration difficulties, and forgetfulness were the major symptoms. The evaluation of her thyroid function did not reveal any abnormality [i.e., thyrotropin (THS) was within the normal range of 0.270–4.200 μU/ml, with a value of 2.54]. A modest improvement was observed during summer and hot weather conditions, but the unpredictable character of the symptoms made the patient feeling insecure and anxious. These had considerable impact on the everyday life, affecting social interaction and professional performance. She appeared healthy when compared with others: being doubted, because of the invisible nature of her pain, had an additional negative impact on the patient’s well-being.

### 2.5 Differential Diagnosis

A deterioration of the patient’s conditions began with a severe worsening of morning stiffness and lower back pain, leading to disabling conditions, and forcing the patient at bed rest with severe aches. This was described as a “torture-like experience,” without any respite for a period of 48 h. The concomitant onset of such a pain to both hips and to the right shoulder led to investigate for rheumatic diseases and to the hypothesis of FMS.

During a first visit of a specialist in rheumatology, the tender points of FMS were assessed (11/18). Blood testing was then performed: rheumatoid factor, anti-nuclear antibodies, anti-nDNA antibodies research, anti-ENA antibodies, and anti-cyclic citrullinated peptide antibodies were all negative. Erythrocyte sedimentation rate was 8 mm/h (normal range: 2–20), C-reactive protein 0.1 mg/dL (normal range: 0.0–0.5), creatine kinase 92 IU/L (normal range: 30–150), and rheumatoid factor 8 IU/mL (normal range: 0–14). Complete blood count was in the normal range.

On a visit by a second specialist in rheumatology, tender points were accessed again, and the differential diagnosis of FMS was made. This was based on the presence of tender point sensitivity (14/18), widespread chronic pain for longer than 3 months, morning stiffness, non-restorative sleep, depression, anxiety, leg and foot cramps, chest pain, tachycardia, hypersensitivity to cold, cognitive impairment as forgetfulness and low concentration, irritable bowel syndrome-constipation (IBS-c), prickling sensations at fingers and toes, bloating, and hyperhidrosis.

The ineffectiveness of pharmacological therapies in FMS came to patient’s knowledge (1). The patient refused the proposed muscle relaxant drug (i.e., tizanidine) on the basis of its unproven effectiveness (1), and she also refused the proposed selective serotonin–norepinephrine re-uptake inhibitor (SNRI) (i.e., duloxetine) (12) on the basis of awareness of collateral effects (13) and development of pharmacological addiction. The patient generally feared collateral effects of the drug treatments, and she was rather interested in the novel metabolic approach for the symptomatic remission in FMS (11).

## 3 Therapeutic Protocol

### 3.1 Guidelines (11)

The therapeutic protocol is a strict diet. It was devised to facilitate Trp absorption, and thus guarantee its bioavailability as a substrate for 5-HT synthesis. In order to sustain 5-HT synthesis, it is mandatory to remove molecules that could negatively affect the fate of Trp in the gastrointestinal tract. The core of this approach is the exclusion of some carbohydrates from the diet and the proper intake of Trp with food (11). Because of fructose is a high reactive sugar (14), limiting the intake of fructose as much as possible is the essential point, including fructose chains, such as fructans and inulins, and some other molecules that do not have specific transport systems (e.g., sorbitol). Glutamate and aspartame should also be excluded (11).

### 3.2 Diet

The patient’s diet includes eggs, meat, fish, clams, potatoes, carrots, celery, spinaches, beets, chards, dark chocolates (at least 70 + % cacao), rice, millet, carob powder, walnuts, extra virgin oil, grape seed oil, thyme, sage, rosemary, coffee, green tea, and small amount of almonds. Almonds, despite containing fructose, still belong to the patient’s diet, as they are well tolerated in small amount, suggested to be consumed together with a glucose source, typically rice or potatoes to activate GLUT2 transporter as remarked in Ref. (11).

Any food, beverage, or herb not in the previous list and not according to treatment guidelines is excluded from the diet protocol. Particularly, processed food containing artificial sweeteners, high fructose corn syrup, sorbitol, glutamate, and aspartame must be excluded: among others soft drinks, fruit juices and the majority of confectionery (11). Food containing free fructose, such as honey and fruits, must be removed from patient’s diet. Most legumes, wheat and most cereals, and many vegetables that contain fructans and inulins (15) must also be removed (11). Attention must also be paid to the excipients in pharmacological preparations, pills, syrups, and solutions (16).

Compared with the previous patient’s diet, the one proposed here does not affect the total daily energy intake (2,200–2,400 kcal/day), but the nutritional profile concerning a reduction in carbohydrates, fibers, and an increase in protein and fat intake. The patient’s diet is thus composed of 31–36% carbohydrates, 30–32% fats, 25–27% proteins, and 9–10% fibers. The previous diet was mainly a Mediterranean diet. It was rich in vegetables, fresh fruits, dried fruit, cereals, and legumes. It contained a moderate amount of fish, meat, dairy products, eggs, nuts, and sweets. Its proportion of nutrients was: 55–56% carbohydrates, 30–32% fats, 17–18% proteins, and 16–18% fibers.

### 3.3 Therapeutic Approach

In order to assess her dietary intake, the patient was asked to keep a food diary. This method requires the subject to list the consumed food and the state of health, reporting the presence of symptoms: widespread pain, fatigue, morning stiffness, bowel function, headaches, sleep quality, cramps, prickling sensation at fingers and toes, mood changes, anxiety, and depressive mood among others. This method allows to evaluate compliance with the diet guidelines and the impact of diet modifications based on symptoms. It makes the patient an active subject to fight against the disease. This approach highly contributes to patient’s motivation and compliance with protocol as it makes the patient conscious of her power on the control of symptoms.

### 3.4 Patient Clinical Response

The growing severity of symptoms highly motivated the patient to strictly follow the diet guidelines. For a complete picture, the patient had already been on a lactose-free diet for 3 years and on a pork meat-free diet for 5 years.

Dietary modifications resulted in a rapid improvement of the patient’s condition after only a few days up to the full resolution of the majority of symptoms in few weeks. Symptoms of depression disappeared. Fatigue unrelieved when rest disappeared and she regained restorative sleep. Chronic musculoskeletal widespread pain and morning stiffness had a marked improvement up to no longer present. She recovered her energy and vitality. She got completely independent in all the activities of her daily life as before the onset of the disease by solving the occupational and social disabilities.

The patient broke the dietary protocol: not admitted foods were arbitrarily, deliberately, and voluntarily assumed (for instance, among others: eating a pear, or a fig, or an onion, or asparagus). It plays as negative control. It is significant for three different reasons: to exclude a major placebo component in the remission of symptoms, to evaluate the short-term effectiveness of the treatment, and to validate the protocol as a final cure or a remission protocol. The recurrence of symptoms is correlated with diet faults. The treatment leads to a remission but it is not a final cure.

Two months after the beginning of the diet the patient was vastly improved in every aspect. She regained her positive mental outlook. She returned to full employment. She recovered her energy and vitality as she did not since years.

### 3.5 Subsequent Course

12 months after the differential diagnosis and 10 months after the beginning of the diet modification the patient is still on diet. Marked not keeping occurred few times, being she well aware of the consequent recurrence of symptoms: when isolated, little faults trigger little symptoms; nevertheless, repeated and continuing faults have the potential for leading to the previous chronic condition of pain, fatigue, and mood symptoms. Moreover, being pain-free, the patient started physical aerobic exercise which she was unable to perform before due to stiffness and widespread musculoskeletal pain. Some comorbidities did not completely solve: sensitivity to cold, hypersensitivity to odors and noise, dysmenorrhea, and memory lapses are still present.

## 4 Discussion

### 4.1 FMS Burden

Fibromyalgia syndrome is really a challenging, insidious, and disabling disease that afflicts patients and their relatives as a real burden in everyday life (10). Epidemiological data clearly demonstrate the socio-economical burden associated with FMS and the urgency of effective answers (4, 10, 17–19). The diagnosis often delayed may exacerbate patient’s conditions. Being doubted due to the invisibility of pain is perceived as a “double burden.” Unfortunately, it is a common condition among patients (9).

Despite the large number of pharmacological and non-pharmacological clinical trials and studies performed, since nineties, an effective cure still lacks (1). The crucial role of 5-HT in FMS is no more a matter of debate. It has been clearly observed in experimental studies, although still not fully understood in its pathophysiological mechanism. Low levels of 5-HT and/or of its precursor Trp were variably observed in such studies, early during 1990s (20–22) and more recently (7, 23, 24). The introduction of selective serotonin re-uptake inhibitors (SSRIs) and SNRIs as a pharmacological therapy in FMS was the consequence in the clinical practice (3, 6, 25–29). Besides Trp, low levels of other essential aminoacids (30, 31) and altered aminoacid homeostasis (32) have been reported in patients with FMS as compared to the general population: anyway, these findings did not translate into an effective cure (1). Surprisingly, the “2016 Revisions to the 2010/2011 fibromyalgia diagnostic criteria” (33) did not contain any explicit reference to blood testing in this direction.

### 4.2 The Novel Remission Protocol Beyond the State of the Art

In this scenario, where the challenge for physicians and healthcare systems to face FMS is clear and still open (1), we report the first case of controlled remission in FMS following a novel metabolic approach (11). This report shows the crucial role of diet in FMS, and food choice as a key strategy for its management. The marked improvements of the patient’s clinical condition open great perspectives to face up FMS burden giving the patients an effective strategy. Intrinsically, a withdrawal approach avoids the potential side-effects associated with pharmacological therapies [i.e., SSRIs and SNRIs (13, 34–36), muscle relaxants, and the interactions among them]. The effectiveness-to-cost ratio of this approach is evident. It is a low risk and accessible therapeutic approach with virtually no costs for the treatment itself, than those related to possible vitamin and mineral salt supplements, and blood testing to evaluate their levels. The economic perspective could be relevant bearing in mind the significant number of patients.

Dietary modifications in FMS are not a new approach: different diets were attempted in the past, variably focused on the elimination of certain food or chemical additives (37). Nevertheless, the therapeutic approaches proposed till now often did not ground on a solid theory which is able to fully predict and explain the experimental outcomes.

### 4.3 The Possible Role of the Placebo and Nocebo Effects

As in any therapeutic approach implemented for chronic pain, a significant placebo response should be considered. The placebo effect is reported in FMS (38, 39). Breaking the diet protocol with not admitted food aims to exclude the remission of symptoms by a main placebo contribution. Although the placebo component could not be excluded at all *a priori*, the occurrence of an *ad hoc* nocebo effect precisely correlated with diet faults (i.e., voluntary breaks of protocol guidelines and accidental mistakes) is highly improbable.

### 4.4 Diet Management and Implementation in the Clinical Practice

It is already known that nonimmunologically mediated adverse reactions to food, which resolved following dietary elimination, are then reproduced by food challenge (40). Clinical improvement was reported after dietary treatment for fructose malabsorption in irritable bowel syndrome (IBS) patients by different studies (41, 42); particularly, a significant reduction of symptoms and improvements in the quality of life proportionate to the amount of eliminated fructose was reported by Choi et al. (43). The human capacity for fructose absorption is widely variable (44); incomplete fructose absorption can occur with doses as low as 5 g in individual considered as health subjects (45). Some authors report that patients with IBS associated with fructose malabsorption can tolerate 10–15 g of fructose per day (46). It is reasonable to suppose that a threshold exists in patients with FMS too, and that the tolerated amount of fructose and of the other not admitted molecules could be related to the severity of the patient’s conditions. The threshold can be very low: the patient reports that even very low amounts of free fructose are able to trigger the symptoms. In severe conditions, a compromise could not be possible at all, and a complete fructose-free diet is the suggestion. A patient-to-patient tailored approach is the best implementation in the clinical practice.

This report supports the protocol intrinsically effective for the remission of symptoms in FMS. It is a matter of fact that adherence to the protocol is correlated with symptomatic improvements and non-adherence with the recurrence of symptoms.

Because of the abundance of fructose in our food supply (as it is present not only in the form of simple monosaccharide, but also in the form of fructose chains), a strict fructose-free and fructan-free diet is binding, and maybe not required once patients experience sufficient relief from their symptoms. Particularly, in not severe conditions, a “re-introduction phase” could be approached by introducing into the diet small amounts of not admitted food, one at a time, in order to determine exactly how much fructose and the other not admitted molecules can be tolerated, to have the least restrictive diet, while keeping symptoms under control. This way, partial compliance with protocol guidelines may be a personal compromise to control the symptoms to a satisfactory level, while minimizing the social limitations that dietary restrictions impose.

The co-ingestion of glucose could be in principle beneficial to allow the presence of small amount of fructose in the diet (11). As previously mentioned, it activates the GLUT2 co-transporter, reducing the non-absorbed fructose (47, 48).

The difficulty of adhering to such restricted diet might be the criticism of it. Nevertheless, compliance with protocol guidelines demonstrates a high symptomatic improvement, being this way an incentive to be on diet. Diet modifications, that improved health condition in an IBS cohort of patients, reveal the willingness and the ability of patients to maintain dietary restrictions to avoid painful meal-related events (49). Patient’s dietary education contributes to the compliance with diet too, so that proper training and active involvement in the way to remission are crucial for successful and durable results.

## 5 Conclusion

This report shows a remarkable clinical improvement in FMS by a strict exclusion diet: remission of depression, pain, stiffness, chronic fatigue, and non-restorative sleep. This result opens new perspectives in the treatment of FMS solving the substantial functional limitations experienced by patients, virtually requiring no costs for the treatment itself. It could be an important point to be considered by healthcare systems, because of the incidence of FMS. The costs associated with the treatment are related to blood investigations and supplements of vitamins and mineral salts in case of deficiencies. Moreover, a dietary strategy has intrinsically no side-effects associated with pharmacological therapies.

A preliminary patient’s training should be considered, in order to gain proper knowledge of the protocol guidelines; it is important that patients can correlate the voluntary or chance failures to the recurrence of symptoms in order to avoid the gate of the “positive feedback loop.”

Because of its efficacy on symptoms, the absence of drug side-effects and its low cost, this approach could be an effective and accessible answer to the burden of FMS, at least to give patients a respite. A pilot study is required to ground this metabolic approach in FMS, and to finally evaluate its inclusion in the guidelines for clinical management.

## Ethics Statement

The subject described in this report has given written informed consent for publication of the above-mentioned data on her FMS case.

## Author Contributions

SML designed the report and wrote the final draft. FI followed the patient in her clinical history. SML and FI collected the patient’s clinical data and approved the final draft for publication.

## Conflict of Interest Statement

The authors declare that the research was conducted in the absence of any commercial or financial relationships that could be construed as a potential conflict of interest.
